# Significant Association between Microrna Gene Polymorphisms and Type 2 Diabetes Mellitus Susceptibility in Asian Population: A Meta-Analysis

**Published:** 2020-05

**Authors:** Zhifang DENG, Wenqi GAO, Wei LUO, Li AI, Min HU

**Affiliations:** 1. Department of Pharmacy, The First College of Clinical Medical Science, China Three Gorges University & Yichang Central People’s Hospital, Yichang, 443000, China; 2. Department of Pharmacy, The Central Hospital of Wuhan, Tongji Medical College, Huazhong University of Science & Technology, Wuhan, Hubei, China; 3. Institute of Maternal and Child Health, Wuhan Children’s Hospital, Tongji Medical College, Huazhong University of Science & Technology, Wuhan, 430000, China; 4. Department of Central Experimental Laboratory & Yichang Key Laboratory of Ischemic Cardiovascular and Cerebrovascular Disease Translational Medicine, The First College of Clinical Medical Science, China Three Gorges University & Yichang Central People’s Hospital, Yichang, 443003, China

**Keywords:** microRNAs, Gene polymorphism, Type 2 diabetes mellitus, Susceptibility

## Abstract

**Background:**

The gene polymorphisms in microRNA might relate to susceptibility of type 2 diabetes mellitus (T2DM). However, the results of existing studies were inconsistent and obscure. To investigate the precise associations between microRNA gene polymorphisms and T2DM risk, the present meta-analysis was performed.

**Methods:**

The literatures were searched from four electronic databases, PubMed, Embase, CNKI and Wan-fang. Subsequently, odds ratios (ORs) and the corresponding 95% confidence intervals (CIs) were both used to evaluate the associations between two single nucleotide polymorphisms (SNPs) (microRNA146a rs2910164 (G>C), microRNA124a rs531564 (C>G)) and risk of T2DM in Asian population.

**Results:**

Totally, there were 4 studies included in our present analysis in the language of English and Chinese. There were partly significant associations between susceptibility of T2DM and SNPs (microRNA146a rs2910164 (G>C), microRNA124a rs531564 (C>G)). The G allele in microRNA146a rs2910164 (G>C) and C allele in microRNA124a rs531564 (C>G) both presented remarkably reduced risk of T2DM when compared with the healthy population.

**Conclusion:**

The microRNA146a rs2910164 (G allele) and microRNA124a rs531564 (C allele) might function as protective factors in the pathogenetic process of T2DM in Asian population.

## Introduction

Type 2 diabetes mellitus (T2DM), characterized by chronic hyperglycemia, is the most common endocrine and metabolic disease among western and more recently, Asian countries. With the development of global economic and changes of lifestyle, the global number of T2DM patients will increase 50.7% by 2030, up to 552 million. Chronic diabetes-associated comorbidities, including cardiovascular disease, kidney disease, and blindness, damaged multiple organs and resulted in high fatality rate. Moreover, T2DM brings out many serious impacts on patients’ mental health and triggers heavy economic burden on patients’ families and society (1). Therefore, T2DM has become a harmful public health problem. It is imminent for us to look for T2DM risk factors and prevent the occurrence and development of it (2). The environmental risk factors, such as age, less physical activity, dietary factors and early nutrition status, are frequently influenced by individual genetic susceptibility. In addition, the high conformance rate (96%) in twin studies indicates that genetic factors significantly contribute to T2DM (3, 4). Thus, genetic predisposition attracts more and more attention of researchers.

MicroRNA is a class of endogenous small noncoding, single strand, and functional RNA, which consist of 21–23 nucleotides with evolutionary conservative sequences. MicroRNA plays an important role in various biological processes of individual development, tube formation, cell proliferation, differentiation and apoptosis by specific complementary base-pairing to the 3′ untranslated region (UTR) of the target mRNA (5, 6). Recent studies show that function of microRNA may influence the development of T2DM, as well as regulate the process of insulin generation and secretion, islet development and function, and lipid metabolism (7–9).

Single-nucleotide polymorphism (SNP) is the most common type of DNA sequence deviations and reportedly account for approximately 90% of genetic variations in the human genome (10). In aspect of microRNA, SNP or gene mutation in microRNA sequence may affect the mature process of pre-microRNA, combination of transcription factors and microRNA, and interaction of target genes and microRNA, ultimately promoting the occurring and development of disease (11, 12). Thus, the SNP in microRNA may possesses various potential functions in affect the susceptibility of disease.

Gene polymorphisms in microRNA were associated with several diseases, including various cancers and metabolic diseases (13–16). These SNPs have been associated with the susceptibility of T2DM, but the results were inconsistent. Therefore, we conducted this meta-analysis to evaluate the relation between gene polymorphisms in microRNA and risk of T2DM in Asian population.

## Methods

### Identification and eligibility of relevant studies

PubMed, Embase, CNKI and Wanfang databases were used to search for all eligible studies. We used the keywords and subjects both in Chinese and English as follows: “microRNA or miRNA”; “polymorphism or variant or mutation or SNP”; “diabetes mellitus or type 2diabetes mellitus or T2DM”. All literatures included were published before May, 2017. Furthermore, if there were multiple investigations focus on the same issue, the nearest and the most integrated investigation was only included in our present neta-analysis. We assessed each potentially eligible study, respectively.

### Inclusion and Exclusion Criteria

We used the following the inclusion criteria to search and obtain the eligible studies. Firstly, the investigation must be case-control study or cohort studies; Secondly, the eligible studies should investigate the association between risk of T2DM and microRNA gene polymorphisms; Thirdly, the eligible studies should possess appropriate and complete description of genotype distribution data in the T2DM patients and control population; The last one, the eligible studies should conduct in regard to Asian population. In addition, the studies on animals, case reports, reviews, abstracts, editorial comments, reports, and with incomplete genotype distribution data were excluded.

### Data Extraction and Quality Assessment

Two investigators red, reviewed, and extracted effective information from literatures that met the inclusion criteria, respectively. The extracted information were consisted of the first author’s name, year of publication, regions of participants (such as Chinese or Iranian), ethnicity, genotyping data, and total number of cases and controls. The present meta-analysis was performed according to the PRISMA statement, which was the best report on system review and meta-analysis.

### Statistical analysis

The Hardy-Weinberg Equilibrium (HWE), representing departure of the frequency of microRNA polymorphism, was assessed by chi-square test or extracted from original studies. There was a significant disequilibrium when *P*<0.05. ORs and corresponding 95% CIs were used to estimate the strength of association between microRNA gene polymorphism and risk of T2DM. *P*<0.05 was indicated that there was a significant difference. We investigated the association between microRNA gene variant and risk of T2DM under five genetic models: additive, the dominant, the recessive, the homozygote, and the heterozygote model. Heterogeneity was evaluated by Chi-square-based Q statistic test and I^2^-statistics, significance was set at *P*<0.05. Fixed effects model (Mantel–Haenszel method) was used when there was no significance in heterogeneity (*P*>0.05), otherwise the random effects model (Dersimonian–Laird method) was chose. Funnel plots was used to access the potential publication bias by the method of Egger’s linear regression test. All data were analyzed by Review Manager (ver. 5.0.0, The Cochrane collaboration), using 2 side *P*-values.

## Results

### Study characteristics

**Fig. 1:**
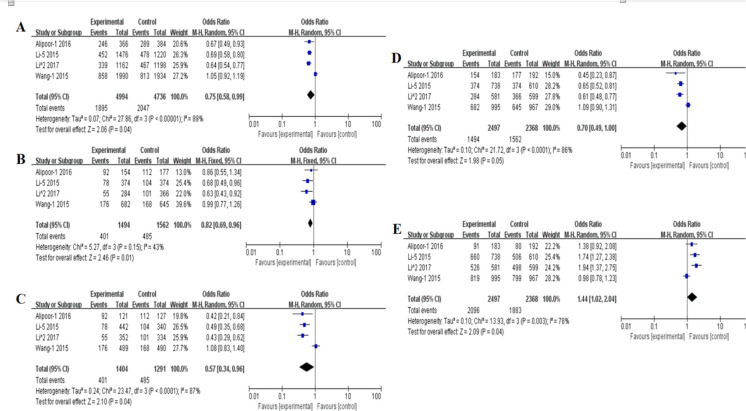
Forest plot for the association between microRNA-146a rs2910164 G>C polymorphism and T2DM (A) Allele model, G vs C, random model. (B) Heterozygote model, GG vs GC, fixed model. (C) Homozygote model, GG vs CC, random model. (D) Recessive model, GC+CC vs GG, random model. (E) Dominant model, GG+GC VS CC, random model

Search process of articles was showed in Fig. 1. There were 176 papers relevant to the search strategy. After screen all the articles, 104 articles were excluded because of irrelevant title and abstract. In the rest 72 articles, 67 were excluded for incomplete genotype data, duplicated data, or other inclusion criteria. One article was excluded because of no Asian population data. Finally, four articles were included in the current meta-analysis, which contained the population of Chinese Han and Iranian. Details of the included studies could be found in Table 1.

**Table 1:** Association of microRNA Polymorphisms with T2DM risk

***First Author***	***Tear***	***Ethnicity***	***microRNA***	***SNP***	***Genotype***	***T2DM***			***Control***			***HWE***
Li^1	2017	Chinese	microRNA-146a	rs2910164	GG/GC/CC	55	229	297	101	265	233	*P*>0.05
Li^2	2017	Chinese	microRNA-124a	rs531564	CC/CG/GG	13	137	431	25	164	410	*P*>0.05
Alipoor	2016	Iranian	microRNA-146a	rs2910164	GG/GC/CC	92	62	29	112	65	15	*P*>0.05
Li-1	2015	Chinese	microRNA-146a	rs2910164	GG/GC/CC	78	296	364	104	270	236	*P*>0.05
Li-2	2015	Chinese	microRNA-124a	rs531564	CC/CG/GG	17	174	547	26	165	419	*P*>0.05
Wang-1	2015	Chinese	microRNA-146a	rs2910164	GG/GC/CC	176	506	313	168	477	322	*P*>0.05
Wang-2	2015	Chinese	microRNA-124a	rs531564	CC/CG/GG	23	291	681	21	257	689	*P*>0.05

### Part association between microRNA-146a rs2910164 (G>C), microRNA-124a rs531564 (C>G) gene polymorphism and T2DM in Asian people

**Fig. 2:**
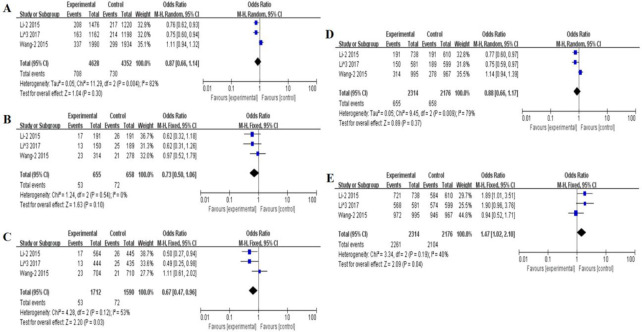
Forest plot for the association between microRNA-124a rs531564 C>G polymorphism and T2DM A: Allele model, G vs C, random model. B: Heterozygote model, GG vs GC, fixed model. C: Homozygote model, GG vs CC, random model. D: Recessive model, GC+CC vs GG, random model. E: Dominant model, GG+GC VS CC, random model

As shown in Figs. 1 and 2, there were partly significant associations between microRNA-146a rs2910164 (G>C) and microRNA-124a rs531564 (C>G) gene polymorphisms and risk of T2DM. There were remarkable associations between microRNA-146a rs2910164 (G>C) and risk of T2DM. Significant statistic differences were observed under all 5 genetic models: G vs. C (OR=0.75, 95%CI: 0.58–0.99), GG vs. GC (OR=0.82, 95%CI: 0.69–0.96), GG vs. CC (OR=0.57, 95%CI: 0.34–0.96), GG+GC vs. CC (OR=0.70, 95%CI: 0.49–1.00), GC+CC vs. GG (OR=1.44, 95%CI:1.02–2.04) (Fig. 1).

Part significant associations were also existed in microRNA-124a rs531564 (C>G) and susceptibility of T2DM. Compared with CC genotype, significantly increased risk of T2DM might be presented in GG (CC vs. GG: OR=0.67, 95%CI: 0.47–0.96) and GC+GG carriers (GC+GG vs. CC: OR=1.47, 95%CI: 1.02–2.10) (Fig. 2).

### Publication Bias

**Fig. 3:**
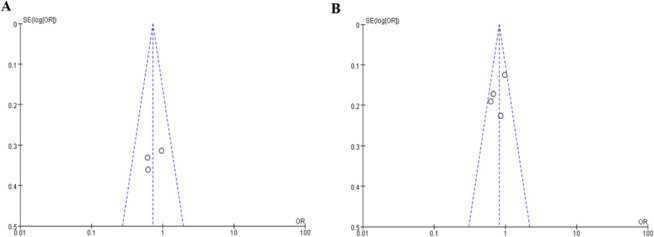
Funnel plot for the association between microRNA-146a rs2910164 G>C (A), microRNA-124a rs531564 C>G (B) polymorphism and T2DM

We used Begg’s funnel plot and Egger’s test to estimate publication bias. There was no significant obvious asymmetry in a funnel plot shape (Fig. 3).

## Discussion

Recent epidemiological studies indicate that the prevalence of T2DM has increased to 11.7% in China. The total number of adult diabetes has increased to 415 million by 2015 (17). Large number of evidences indicate that T2DM is a multifactorial disease, genetic component play a strong role in the pathogenetic process of T2DM. Recently, microRNA gene polymorphisms served a functional role for susceptibility to T2DM (18–20). However, the existing results were inconsistent. Therefore, we drawed current meta-analysis in order to derive a more precise estimation of the relationship.

Our present meta-analysis performed for the first time to assess the relationship between the susceptibility of T2DM and microRNA gene polymorphisms in Asian population. Totally 4736 controls and 4994 T2DM patients in Chinese Han and Iranian were included in our investigation. The results of microRNA-124a rs531564 (C>G) showed that models of CC VS GG and GC+GG VS CC were protective factors in developing T2DM. In the analysis of microRNA-146a rs2910164 (G>C), the 95% CIs in all 5 genetic models were below 1, and *P*-value were all less than 0.05, suggesting that there was obvious statistical significance between this SNP and risk of T2DM. Moreover, the results indicated people who carrying G allele might predict lower risk of T2DM. Of interest, our results was different with Wang (20). The limited number of literatures and participants might be the primary reason for this inconsistency. Thus, microRNA mimics or inhibitors directly regulating microRNA expression might function as the novel and promising therapeutic targets.

In addition, multiple limitations were present in the present analysis. 1) We performed this analysis in Asian population, however, the ethnicity of most subjects was Chinese Han people and only one study involving in Iranian people included, which made restriction that the general application of the results to other Asian population; 2) The studies included in our research were only published in Chinese and English, not other language, therefore, we might miss partial data; 3) to date, only 4994 T2DM patients and 4739 controls were available for these two microRNA gene polymorphisms, the relatively small size of subjects including in this study made it difficult to perform stratified analyses; 4) many other factors might also take part in the pathogenetic process of T2DM, including environment, gender, etc. The SNPs cannot represent all the risk factors of T2DM.

## Conclusion

The microRNA-146a rs2910164 (G>C) and microRNA-124a rs531564 (C>G) possessed obvious relationships to T2DM. G allele in microRNA-146a rs2910164 (G>C) and models of CC VS GG and GC+GG VS CC in microRNA-124a rs531564 (C>G) might be beneficial alleles and decrease the risk of T2DM, which indicating these SNPs might be served as targets for treatment of T2DM. However, more and more eligible studies with rigorous design were needed to further confirm the associations between microRNA gene polymorphisms and risk of T2DM.

## Ethical considerations

Ethical issues (Including plagiarism, informed consent, misconduct, data fabrication and/or falsification, double publication and/or submission, redundancy, etc.) have been completely observed by the authors.
